# Light Activates Output from Evening Neurons and Inhibits Output from Morning Neurons in the *Drosophila* Circadian Clock

**DOI:** 10.1371/journal.pbio.0050315

**Published:** 2007-11-27

**Authors:** Marie Picot, Paola Cusumano, André Klarsfeld, Ryu Ueda, François Rouyer

**Affiliations:** 1 Institut de Neurobiologie Alfred Fessard, CNRS UPR 2216, Gif-sur-Yvette, France; 2 Invertebrate Genetics Lab, Genetic Strains Research Center, National Institute of Genetics, Mishima, Shizuoka, Japan; University of Geneva, Switzerland

## Abstract

Animal circadian clocks are based on multiple oscillators whose interactions allow the daily control of complex behaviors. The *Drosophila* brain contains a circadian clock that controls rest–activity rhythms and relies upon different groups of PERIOD (PER)–expressing neurons. Two distinct oscillators have been functionally characterized under light-dark cycles. Lateral neurons (LNs) that express the pigment-dispersing factor (PDF) drive morning activity, whereas PDF-negative LNs are required for the evening activity. In constant darkness, several lines of evidence indicate that the LN morning oscillator (LN-MO) drives the activity rhythms, whereas the LN evening oscillator (LN-EO) does not. Since mutants devoid of functional CRYPTOCHROME (CRY), as opposed to wild-type flies, are rhythmic in constant light, we analyzed transgenic flies expressing PER or CRY in the LN-MO or LN-EO. We show that, under constant light conditions and reduced CRY function, the LN evening oscillator drives robust activity rhythms, whereas the LN morning oscillator does not. Remarkably, light acts by inhibiting the LN-MO behavioral output and activating the LN-EO behavioral output. Finally, we show that PDF signaling is not required for robust activity rhythms in constant light as opposed to its requirement in constant darkness, further supporting the minor contribution of the morning cells to the behavior in the presence of light. We therefore propose that day–night cycles alternatively activate behavioral outputs of the *Drosophila* evening and morning lateral neurons.

## Author Summary

Living organisms have evolved circadian clocks that anticipate daily changes in their environment. Their clockwork is fully endogenous, but can be reset by external cues. (Light is the most efficient cue.) The circadian neuronal network of the fruit fly (*Drosophila*) brain perceives light through the visual system and a dedicated photoreceptor molecule, cryptochrome. Flies exhibit a bimodal locomotor activity pattern that peaks at dawn and dusk in light–dark conditions. These morning and evening activity bouts are controlled by two distinct neuronal clocks in the fly brain. By using flies with a deficient cryptochrome pathway, we have uncovered an unexpected role for light in the circadian system. In addition to synchronizing the two oscillators to solar time, light also controls their behavioral output. The morning oscillator can periodically rouse the fly when in constant darkness, but not in constant light, whereas the evening oscillator can do the same in constant light, but not in constant darkness. This suggests the existence of a light-dependent switch between oscillators that appears to require the visual system. Such a mechanism likely contributes to better separate the active periods of the fly at dawn and dusk, and may help the animal to adapt to seasonal changes in day length.

## Abstract 3

In fruit flies, light not only resets the circadian clock to solar time, but also enables the signaling from one oscillator while disabling the signaling from the other.

## Introduction

Circadian rhythms are controlled by endogenous clocks that tick with an approximately 24-h period fitted to the rotation of the earth. They are synchronized to day–light cycles by environmental cues, the strongest of which is light. Since activity must occur at the most favorable time of the day, the rest–activity rhythm is one of the most tightly clock-controlled behaviors. In natural conditions, many animal species display bimodal rest–activity profiles with activity peaks that anticipate dawn and dusk, and adjust to seasonal changes in day length [1,2]. A similar activity pattern is observed in laboratory light–dark (LD) conditions. A lengthening of the light episode induces a morning-peak advance and an evening-peak delay in mice, suggesting the existence of morning and evening oscillators in the mammalian brain that contribute to seasonal adaptation [3]. The cellular basis of such a dual oscillator has not been characterized in mammals, but has been recently described in *Drosophila*.

The *Drosophila* behavioral clock rests upon approximately 150 neurons that express the PERIOD (PER) protein, divided into three lateral and three dorsal groups, as well as a recently described lateral-posterior group [4–6]. The lateral neurons (LNs) can be divided into cells that express the pigment-dispersing factor (PDF) neuropeptide, and PDF-negative cells. The PDF-expressing cells are four to five large ventral lateral neurons (l-LN_v_s) and four small ventral lateral neurons (s-LN_v_s), whereas the PDF-negative cells are a single s-LN_v_ (the fifth s-LN_v_) and six dorsal lateral neurons (LN_d_s). In LD cycles, PER expression in the four PDF-expressing s-LN_v_s is sufficient to drive activity that anticipates lights-ON, and hence these cells contain a morning oscillator (MO), whereas the addition of four PDF-negative LNs (fifth s-LN_v_ plus three LN_d_s) is sufficient to drive lights-OFF anticipation; hence, the latter cells contain an evening oscillator (EO) [7]. Another group reported similar results [8]. They additionally indicated that dorsal neurons (DNs) could contribute to both the MO and EO. We will therefore specifically refer to the morning oscillator residing in PDF-positive LNs as the LN-MO and to the evening oscillator residing in the PDF-negative LNs as the LN-EO. We have previously shown that, in constant darkness (dark–dark; DD), clock function restricted to the LN-MO is sufficient to generate robust 24-h activity rhythms, whereas clock function in the LN-EO is not [7]. This suggested that, in the absence of light, the LN-MO is the driving oscillator of the circadian network. Indeed, it has been shown that at least part of the LN-EO behaves in DD as a driven oscillator, reset by the LN-MO in each circadian cycle [9].

Circadian clocks are very sensitive to light and respond to it in different ways. First, light is the main clock synchronizer, and LD cycles entrain the *Drosophila* brain clock through two separate light-input pathways. The blue-light–sensitive protein cryptochrome (CRY) is present in most clock neurons [10,11]. Light-activated CRY binds to the TIMELESS (TIM) protein and induces its degradation, which is likely to reset the molecular oscillator [12–15]. *cry^b^* mutants do not respond to short light pulses and fail to quickly resynchronize to a shift of the LD cycle [10,11,16–18]. The *cry^b^* mutation is located in the flavin-binding domain and certainly abolishes CRY photoreceptive function [17]. Although the CRY^b^ protein is barely detectable by anti-CRY antibodies [17], very low amounts may still be present and play some non-photoreceptive function in the mutants. The visual system, which includes the compound eye and the extra-retinal Hofbauer-Büchner eyelet, provides additional rhodopsin-dependent light inputs to the brain clock [19–21]. They are not sufficient for clock responses to short light pulses, but allow entrainment by LD cycles (although less efficiently than CRY). Only flies depleted for both functional CRY and the visual system appear circadianly blind [11,18].

Besides entrainment, light affects other parameters of the circadian system, including its internal synchrony as well as the robustness of the rhythm and its period [22]. In constant light (light–light; LL), wild-type flies become arrhythmic, whereas *cry^b^* mutants retain robust rhythmicity with a 24–26-h period [11,14,15,18,23,24], presumably because the absence of functional CRY prevents the light-induced disappearance of TIM in the mutants. Indeed, mutations affecting the CRY-dependent degradation of the TIM protein also produce robust 24–26-h activity rhythms in LL [24,25]. Two studies reported that *cry^b^* mutants display split rhythms in LL, with a major long-period (∼25 h) component and a minor short-period (∼22.5 h) one [26,27]. These slow and fast components appear to correlate with molecular oscillations in some of the PDF-negative LNs and in the PDF-positive s-LN_v_s, respectively [27], suggesting that they may originate from these subsets. Genetic background, light specifications, and behavioral setup are likely to influence splitting occurrence, but the main reason why split rhythms have only been observed in these two studies is likely related to their longer activity recordings, since split components usually appear after several days in LL [26,27].

The present work is aimed at understanding how the previously defined LN-MO and LN-EO control rhythmic behavior in the presence or the absence of light. We have generated flies that were mosaic with respect either to CRY signaling or to the presence of a functional clock. In particular, we altered functional CRY levels separately in either PDF-positive or PDF-negative neurons. We similarly restored PER expression in *per^0^;; cry^b^* double mutants only in precisely targeted neurons. The results indicate that light has opposite effects on the LN-MO and LN-EO, activating the rhythmic behavioral output induced by the evening cells and inhibiting the rhythmic behavioral output induced by the morning cells. Surprisingly, we found that light acts downstream from the molecular clock, since the behavior, but not the molecular oscillations, is light-dependent. We also show that *cry^b^ pdf^0^* double mutants are rhythmic in LL, further supporting the light-induced preeminence of PDF-negative cells.

## Results

### PER Oscillations in the LN-MO and LN-EO in Constant Light

We first analyzed PER oscillations in *cry^b^* mutants in LL, under conditions in which split behavioral rhythms do not occur (see Materials and Methods). As previously described (see above), the mutants displayed a slightly lengthened period (Tables 1 and S1). In *cry^b^* brains dissected on the third day in LL, the PDF-positive s-LN_v_s and some PDF-negative LNs showed PER cycling, whereas the l-LN_v_s and three subsets of DNs did not (unpublished data). This is very similar to the molecular oscillations described by Rieger et al. [27] for *cry^b^* mutants in LL, before splitting would eventually occur. Since PER cycling in LL appeared to be restricted to the PDF-positive and PDF-negative LNs, we decided to focus our study on these groups of clock neurons. In addition, we decided to center the study upon the effect of light on the rhythmicity of the two LN oscillators, and we voluntarily put aside the role of cryptochrome and the visual system in their entrainment pathways.

**Table 1:**
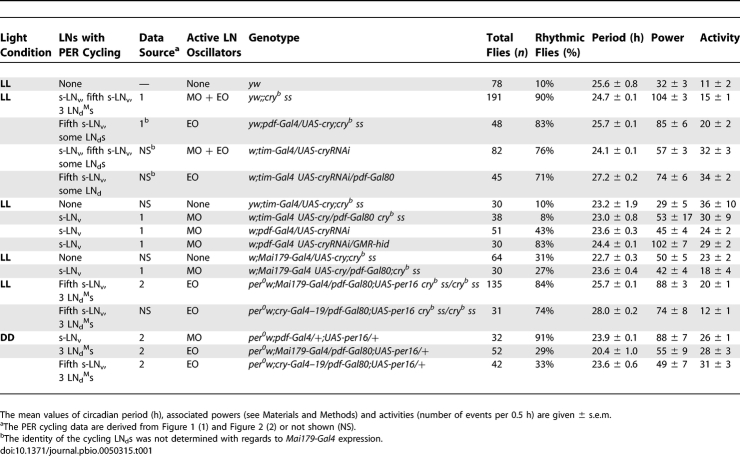
Locomotor Activity Rhythms

**Figure 1:**
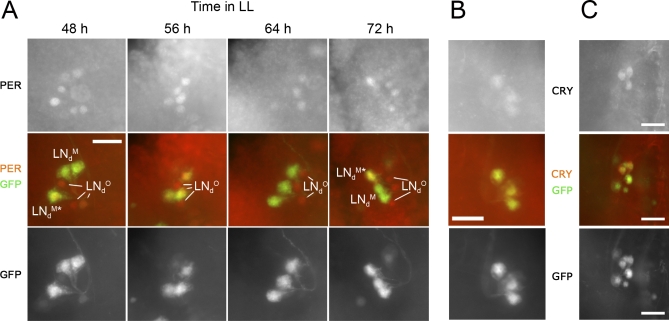
Characterization of Different Subgroups of LN_d_s (A) PER immunoreactivity in LN_d_s of *cry^b^* mutants during the third day in LL. Top row: PER labeling; bottom row: GFP labeling; and middle row: merged. *w;Mai179-Gal4/UAS-gfp;cry^b^ ss* flies were entrained for 4 d in LD conditions (20 °C) before transfer to LL. PER staining was performed on 15 to 20 brain hemispheres for each time point. At 48 and 72 h in LL, one of the *Mai179-Gal4*–positive cells (LN_d_^M*^) is strongly labeled, whereas the other two (LN_d_^M^s) are either not visible (as shown here at 72 h) or only weakly labeled (as shown here at 48 h). At intermediate time points, PER labeling is much more homogeneous in the three *Mai179-Gal4–*positive LN_d_s, being either moderate (56 h) or almost completely absent (64 h). For the quantization of PER expression shown in Figure 2A, the three *Mai179-Gal4–*positive LN_d_s were thus treated as a single group at time points when no clear difference in labeling was observed between them (56, 60, and 64 h). The three *Mai179-Gal4*–negative LN_d_s (LN_d_^O^s) display similar PER labeling at all time points. (B and C) CRY immunoreactivity in wild-type LN_d_s. Top row: CRY labeling; bottom row: GFP labeling; and middle row: merged. CRY staining was performed on (B) *w;Mai179-Gal4/UAS-gfp* brains (after flies had been left in the dark for 4 d) or (C) *w;cry-Gal4–39/UAS-gfp* brains (after flies had been left in the dark for >8 d). CRY labeling is always observed in only three LN_d_s, which are the *Mai179-Gal4–*positive ones. Scale bars indicate 20 μm.

**Figure 2:**
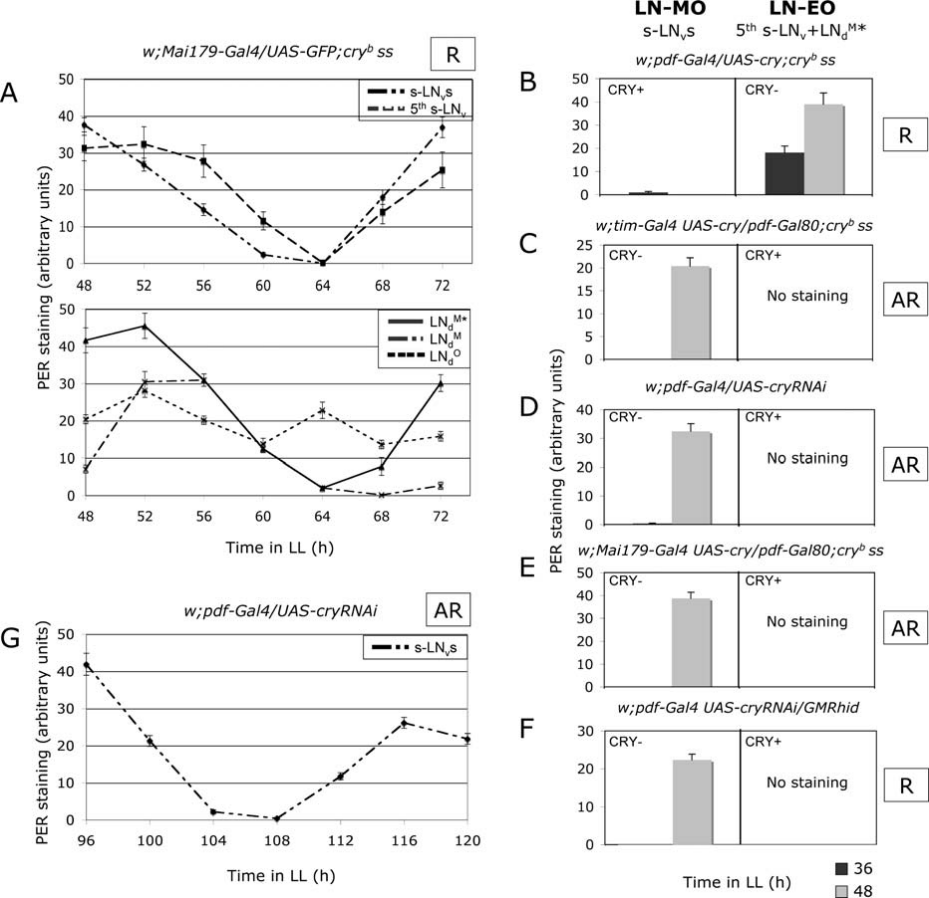
PER Oscillations in the LN-MO and LN-EO Neurons in Constant Light Fly entrainment and PER quantification were performed as described in Figure 1. Error bars represent the s.e.m. for each LN subset. Behavioral rhythmicity in LL (see Table 1) is reported on the right of each genotype (R: rhythmic, and AR: arrhythmic). Unmentioned neuronal groups do not show PER cycling. (A) Brains were dissected during the third day in LL. Top panel: PER levels in the four PDF-expressing s-LN_v_s and the fifth s-LN_v_. Bottom panel: PER levels in the three types of LN_d_s (one *Mai179-Gal4–*positive LN_d_^M*^ + two LN_d_^M^s and *Mai179-Gal4*–negative LN_d_^O^s, as shown in Figure 1A). LN_d_^M*^ is the previously described extra LN_d_ [27]. (B–F) brains were dissected after 36 h (dark bars) and 48 h (light bars) in LL, corresponding to the expected trough and peak in PER oscillations, respectively. The left part of each panel shows PER oscillations in the LN-MO (PDF-positive s-LN_v_s), and the right part shows PER oscillations in the two most strongly oscillating neurons of the LN-EO (fifth s-LN_v_ + LN_d_^M*^). The presence or absence of CRY is indicated for each neuronal group. PER staining is significantly different between the two time points in each genotype (*p* < 0.0005). (G) Brains were dissected during the fifth day in LL. The panel shows PER oscillations in the LN-MO (PDF-positive s-LN_v_s). No PER oscillations were detected in LN-EO neurons (see [D]).

To first clarify the heterogeneity of the LN_d_s group (see also [27]), we examined the *Mai179-Gal4*–driven green fluorescent protein (GFP) expression profile, which includes the previously characterized LN-EO [7] (see Figure S1). In LL, PER cycling was detected in all four LN-EO neurons (Figures 1A and 2A). The fifth s-LN_v_ and the previously described [27] cycling LN_d_ (called here LN_d_^M*^) displayed the strongest oscillations, but the two other *Mai179-Gal4*–positive LN_d_s (LN_d_^M^s) also showed robust, although slightly delayed, oscillations (trough at circadian time [CT]68 instead of CT64). Conversely, constant PER levels were observed in the three *Mai179-Gal4*–negative LN_d_s (LN_d_^O^s; Figures 1A and 2A). Interestingly, CRY immunoreactivity was detected in the three *Mai179-Gal4*–positive LN_d_s (one LN_d_^M*^ + two LN_d_^M^s), but not in the *Mai179-Gal4*–negative LN_d_^O^s in DD (Figure 1B and 1C). These data strongly support the existence of two LN_d_ subgroups: three *Mai179-Gal4*–expressing CRY-positive cells constituting the LN-EO with the fifth s-LN_v_, and three *Mai179-Gal4-* and CRY-negative cells, whose function is unknown.

We then checked whether the presence of functional CRY affects PER expression in an oscillator-autonomous manner in LL, using the two most strongly cycling *Mai179-Gal4*–expressing PDF-negative LNs as reporters for the LN-EO. The main additional Gal4 lines we used here were *pdf-Gal4* [28] to drive expression in the PDF-positive cells only, and *tim-Gal4* [29] to drive expression in all clock cells. The *pdf-Gal80* transgene was used to inhibit GAL4 activity in the PDF-positive cells, and thus “subtract” their contribution from any wider GAL4-expressing cell ensemble [8]. As in wild-type flies, PER levels remained low or undetectable in all cells that contained functional CRY, and PER oscillations were observed exclusively in some of the cells expressing either strongly reduced CRY levels (through *cry* RNA interference [RNAi]) or the mutated CRY^b^ protein (Figures 2B–2E and S2). The four PDF-positive s-LN_v_s and the two selected PDF-negative LNs displayed oscillations whenever they were made CRY deficient (Figures 2B and S2). We conclude that, when functional CRY is reduced or absent, PER oscillations in LL persist in the previously characterized LN-MO and LN-EO.

### Light Inhibits the Behavioral Output of the LN Morning Oscillator

**Figure 3:**
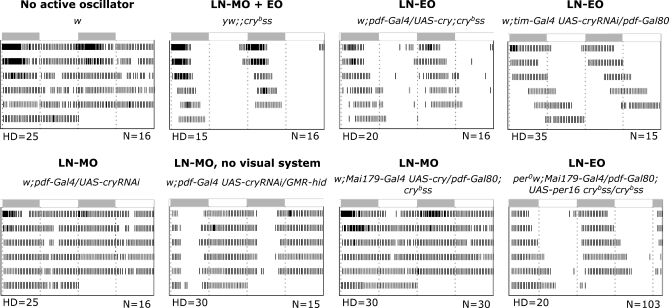
Activity Profiles in Constant Light Average double-plotted actograms during 6 d (top to bottom) of LL after LD entrainment. White and grey bars above each panel indicate entrainment light and dark periods, respectively. Active LN oscillators are reported for each genotype. Hash density (HD) varies according to the genotype for better clarity of the actogram (see Materials and Methods). True activity levels are reported in Table 1. N, number of flies for each genotype.

We then analyzed the behavior of flies with PER oscillations in either the PDF-expressing or the PDF-negative neurons in LL. Genotypes with CRY only (and consequently no PER) in PDF-expressing cells were almost as rhythmic as *cry^b^* mutants (Figure 3 and Table 1; see also Table S1), with a consistently long period. Contrary to DD, the PDF-negative cells can therefore drive behavioral rhythms autonomously in LL, in the absence of any PER oscillations in the PDF-positive LN_v_s. Conversely, flies with CRY only (and consequently no PER) in PDF-negative cells are mostly arrhythmic (Table 1), despite robust PER oscillations in their PDF-positive s-LN_v_s (Figure 2C and 2D). This demonstrates that the four LN-MO neurons cannot drive robust behavioral rhythms autonomously in LL, as opposed to their ability to do so in DD. PER oscillations persist in such flies at least up to the fifth day in LL (Figure 2G), whereas their behavior becomes arrhythmic within the very first days (Figure 3). We thus conclude that in *cry^b^* flies, constant light appears to inhibit the behavioral output of the LN-MO, but not the molecular oscillator itself.

To understand whether light inputs coming from the visual system participate to the LL behavioral rhythms of *cry^b^* flies, we induced its genetic ablation by expressing the apoptotic gene *head involution defective* (*hid*) under the control of photoreceptor-specific regulatory sequences. The *GMR-hid* strain [30] was previously shown to completely lack all visual *glass* gene-dependent structures, but to retain the *glass*-dependent set of DN1s that express PER in the adult brain [11]. *GMR-hid*–induced ablation of the visual system restored the behavioral function of the PDF-expressing neurons in LL, now driving rhythms with a 24.4-h period (Figure 3 and Table 1), although there was no detectable change in PER oscillations (compare Figure 2D and 2F). This indicates that the inhibition of the LN-MO behavioral output by light depends on the visual system.

### Light Is Required for the Behavioral Output of the LN Evening Oscillator

**Figure 4:**
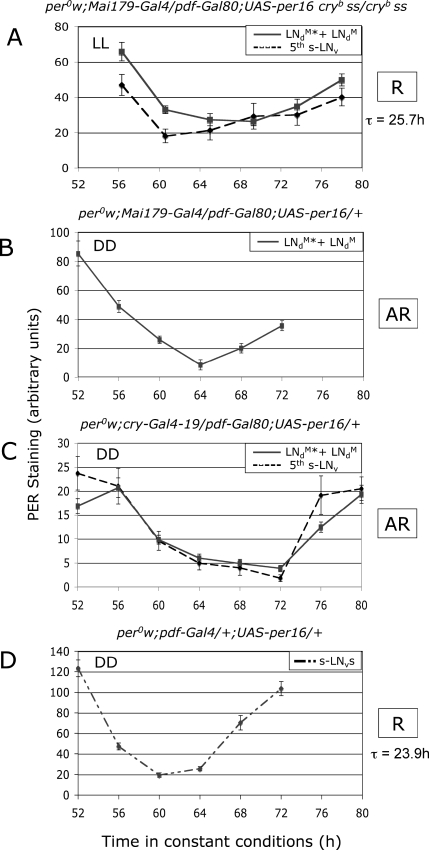
PER Oscillations in LN-MO- or LN-EO-Only Flies in Constant Conditions Fly entrainment and PER quantification were performed as described in Figure 1. Brains were dissected during the third day in LL (A) or in DD (B–D), with time 0 corresponding to the end of the last LD cycle. Dissecting times have been chosen every 4 h for all genotypes except the one with a longer behavioral period in (A), for which time points have been chosen accordingly. Note the delay between the LN-EO neurons in (B and C), and the LN-MO neurons in (D). Behavioral rhythmicity is reported for each genotype (see Table.1). AR, arrhythmic flies; R, rhythmic flies.

What is the neuronal basis of the long-period, LN-MO–independent rhythmicity of *cry^b^* flies in LL? Restoring CRY in only the LN-EO (three LN_d_s plus the PDF-negative fifth s-LN_v_) (Figures 2E and S2) rendered the flies as arrhythmic as the wild type (Figure 3 and Table 1). Thus the LN-EO is necessary for that long-period rhythmicity. Indeed, *per^0^ ;; cry^b^* double mutants with *Mai179-Gal4*–driven PER expression restricted to the LN-EO displayed robust activity rhythms, with a long 25.7-h period (Figure 3 and Table 1). In these LN-EO–only flies, PER levels robustly cycled in all four neurons, displaying a trough after 65 h rather than 60 h in LL, consistent with a period close to 25.5 h rather than 24 h (Figures 4A and S3). A similar behavior was obtained with the *cry-Gal4–19* driver (Table 1), which gives a PER expression pattern very close to *Mai179-Gal4* (Figure S1). The LN-EO is thus not only necessary, but also sufficient to drive rhythmic behavior in LL, whereas it is not sufficient in DD ([7] and Table 1). However, genotypes with PER cycling in the LN-MO neurons in addition to the LN-EO neurons displayed a slightly shorter period than flies with PER in the LN-EO neurons only (Table 1), suggesting that the LN-MO somehow influences the period of the LN-EO and therefore participates in the LN-EO–driven rhythmic behavior. Interestingly, long-period PER oscillations in the LN-EO neurons were observed in DD (trough after 64 h in Figure 4B or between 64 and 72 h in Figure 4C, to be compared with 60 h in Figure4D; see also Figure S3), similarly to LL, although such LN-EO–only flies were behaviorally arrhythmic, contrary to LN-MO–only flies (Table 1). We conclude that in the absence of light, the LN-EO is running at the molecular level, but that its behavioral output is inhibited since it cannot drive activity rhythms.

### PDF Signaling Is Not Required for Robust Rhythms in Constant Light

**Figure 5:**
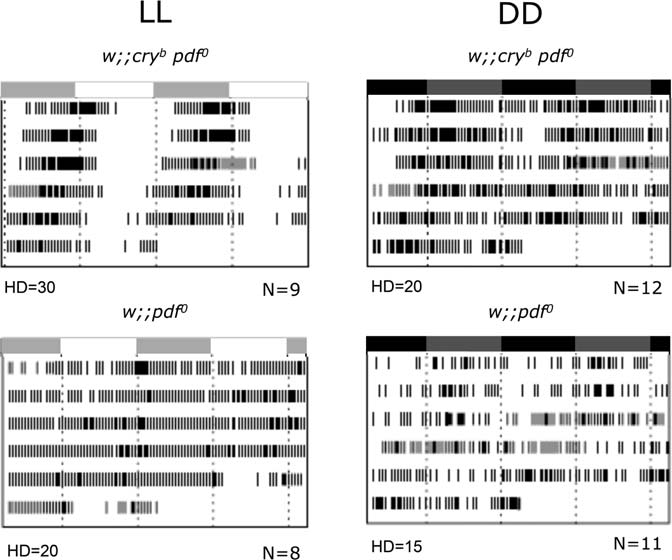
Activity Profiles of *pdf^0^* and *cry^b^ pdf^0^* Flies Average double-plotted actograms during 6 d (top to bottom) of LL or DD after LD entrainment. White and grey (LL) or grey and black (DD) bars above each panel indicate entrainment light and dark periods, respectively. Drawing density varies according to the genotype for better clarity of the actogram, see Table 2 for true activity levels. HD, hash density of the actogram; N, number of flies for each genotype.

Since PDF is required for robust behavioral rhythmicity in DD [28], we asked whether rhythmicity in LL would also depend on PDF signaling. We therefore constructed *cry^b^ pdf^0^* double mutants and tested them in LL. Such flies indeed displayed strong rhythmicity (Figure 5 and Table 2), similar in robustness to that of *cry^b^* mutants (see high power values in Tables 1 and 2), but with a short 22.8-h period. We then analyzed PER oscillations in the double mutants in LL. PER cycling in the EO neurons was in good agreement with the short-period behavior, whereas PER cycling in the MO neurons was not (Figure S4). These data strongly suggest that the EO neurons drive LL activity rhythms in the *cry^b^ pdf^0^* flies, whereas the robustly cycling MO neurons do not contribute significantly to the PDF-independent LL behavior.

**Table 2:**
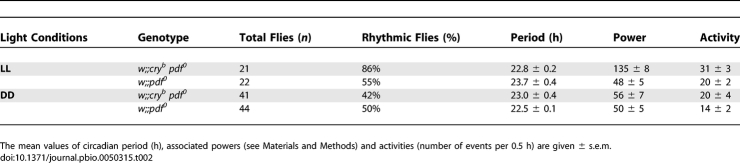
Locomotor Activity Rhythms of *pdf^0^* and *cry^b^ pdf^0^* Flies

We conclude that the LN-EO does not require PDF to generate behavioral rhythms in LL, although PDF strongly influences its period. Conversely, the double mutants were mostly arrhythmic in DD (Table 2), with a fraction of the flies displaying a weak short-period rhythmicity as reported for *pdf^0^* mutants in DD [11,28,31]. The rhythmicity of *pdf^0^* mutants was not improved in LL, showing that the strong rhythmic behavior of the double mutants in LL results from the *cry^b^* mutation.

## Discussion

The PDF-expressing LNs and the PDF-negative LNs were previously characterized as morning and evening cells, respectively, in LD conditions [7,8]. Furthermore, the morning LNs were able to drive robust 24-h rhythms in DD, whereas evening LNs were not [7]. We show in this study that in LL, the evening LNs drive robust rhythms when cryptochrome signaling is absent or reduced, whereas the morning cells are not able to do so. Surprisingly, the molecular oscillations of both groups can be uncoupled from behavioral rhythmicity, depending on light conditions. In DD, the two LN groups show autonomous molecular cycling, but there is no behavioral output when the LN-EO is cycling alone. In LL (and reduced CRY signaling), both groups still show autonomous cycling, but there is no behavioral output when the LN-MO is cycling alone. We therefore conclude that light has opposite effects on the behavioral output of the two LN oscillators, activating it from the evening LNs and inhibiting it from the morning LNs.

The opposite effects of light on the behavioral outputs do not appear to be related to entrainment, since PER oscillations in both the PDF-positive and PDF-negative LNs are synchronized to the LD cycles even in the absence of CRY signaling. The inhibiting effect of light on the LN-MO behavioral output is abolished when the visual system is genetically ablated. This suggests that the projections of the visual system photoreceptors convey, not only input information to the PDF cells (light entrainment), but also signals to control their behavioral output (light inhibition). It is tempting to speculate that light exerts both effects through a direct connection of the PDF cells with the visual system. The Hofbauer-Büchner eyelet photoreceptors that project directly to the LN-MO neurons and participate in the entrainment [19,20] provide a possible pathway.

It was recently reported that the overexpression of PER [32] or of the SHAGGY (SGG) kinase [33] in the PDF-negative clock neurons induced rhythmic behavior in LL. The rhythmicity was associated with the cycling of PER subcellular localization in some of the DNs, whereas the PDF-expressing cells were molecularly arrhythmic. These studies therefore concluded that some DN subsets are able to drive behavioral rhythms in LL. Different groups of PDF-negative cells may thus be able to drive behavioral rhythms in constant light, depending on whether and how the molecular clock is manipulated. Such manipulation could also directly affect molecular oscillations, making them less easy to detect. Since CRY does not appear to have a core clock function in the brain, our data are largely based on situations in which the clock mechanism is little if at all altered. The data support a major contribution of the LN-EO to the robust rhythms of *cry^b^* mutants in LL.

The strong rhythmicity of the *cry^b^ pdf^0^* double mutants in LL contrasts with their weak rhythmic behavior in DD. Altogether, our results strongly suggest that this robust rhythm is generated by the LN-EO, which would therefore behave as a PDF-independent autonomous oscillator. However, the period of the oscillator is clearly influenced by PDF signaling, and thus by the LN-MO, going from 24–25 h in *cry^b^* to 22–23 h in *cry^b^ pdf^0^* flies. An attractive possibility is that the strong short-period rhythm observed in the *cry^b^ pdf^0^* double mutant in LL has the same neuronal origin as the weak short-period rhythm described for *pdf^0^* mutants in DD [28]. The cellular basis of this PDF-independent oscillator in DD remains unclear [11,31,34], although the presence of similar rhythms in flies genetically ablated for the PDF-expressing neurons [28,35] suggests that it originates from other clock cells.

Different results were obtained for the recently described DN-based LL oscillators. When transferred to a *pdf^0^* background, all SGG-overexpressing flies were found to be arrhythmic [33], whereas about 60% of the PER-overexpressing flies displayed long-period rhythms [32]. This suggests that different types of DNs with different sensitivity to PDF may have been analyzed in these two studies. Although some DNs may contribute to the PDF-independent rhythms, our data suggest a strong contribution of PDF-negative LNs to the rhythmic behavior that persists in *pdf^0^* mutants. The weakness of the short-period rhythm of *pdf^0^* flies in DD may reflect the inhibition of the LN-EO output in the absence of light.

**Figure 6:**
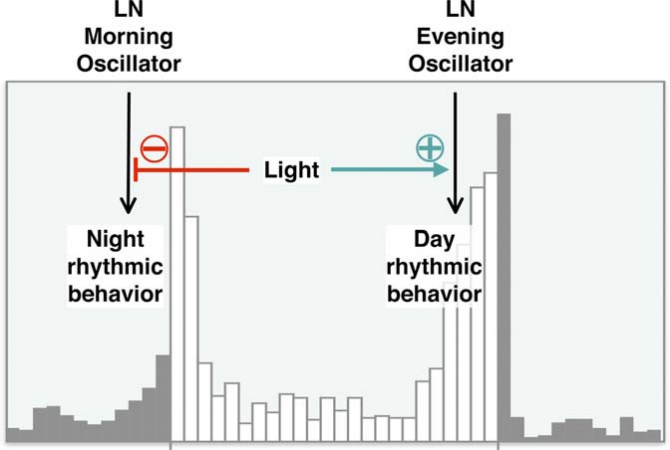
Model for a Light-Induced Switch between LN-MO and LN-EO in LD Conditions In LD cycles, the LN-MO and LN-EO have been shown to produce the morning and evening activity peak, respectively. We propose that light contributes to this bimodal partitioning of activity by negatively controlling the output of the LN-MO while positively controlling the output of the LN-EO, without affecting the molecular oscillators themselves. White bars: light, and grey bars: dark.

Our results indicate that whereas the LN-MO autonomously drives rhythmic behavior in constant darkness, the LN-EO plays this role in constant light, if CRY signaling is abolished or reduced. We thus suggest that in natural LD conditions, *Drosophila* behavior could be driven by the LN-MO during the night, and by the LN-EO during the day, when cryptochrome is quickly degraded by light. This supports a model of a light-induced switch between the circadian oscillators of the LNs (Figure 6) that would allow a better separation of the dawn and dusk activity peaks in day–night conditions. It has been shown that PDF-expressing LNs drive the clock neuronal network in short days, whereas PDF-negative DN subsets take the lead in long days [33]. Our results suggest that the PDF-negative cells of the LN-EO could also be a major player during the long days. Surprisingly, we find that light does not seem to act on the molecular oscillations, but inhibits the LN-MO behavioral output and promotes the LN-EO behavioral output, which may provide an efficient fine tuning of the contributions of the two oscillators. It therefore appears that the visual system controls both the input (entrainment) and the behavioral output of the LN oscillators in the *Drosophila* brain clock. In species such the honeybee or the flour beetle, which appear to lack a light-sensitive CRY protein [36,37], this role of the visual system may be particularly important.

## Materials and Methods

### Fly strains.

The cryRNAi construct produces a double-stranded RNA (dsRNA) that corresponds to the 300–799 region of the cry-RA mRNA (see http://flybase.bio.indiana.edu/reports/FBgn0025680.html). The primers used for PCR were:

5′ primer: AAGGCCTACATGGCCGGACCGATGTGGGTTACAATCGGATGC

3′ primer: AATCTAGAGGTACCGAAGCCCATGTTGTCTCCATA.

The 500-bp DNA fragment was inserted into the pUAST-R57 vector as described here: http://www.shigen.nig.ac.jp/fly/nigfly/about/aboutRnai.jsp.

Two *UAS-cryRNAi* insertions were generated, and the line with the strongest expression (R3) was used in this study. When combined with *UAS-cry* and the *pdf-Gal4 driver*, this *UAS-cryRNAi* insertion reduced CRY levels by at least 80% (unpublished data), as judged by immunocytofluorescence with anti-CRY. The *UAS-cry* [10] and *UAS-per16* [35] insertions have been described previously. *tim-Gal4* is expressed in all clock neurons in addition to several non-clock neuronal groups [29], and *pdf-Gal4* is specifically expressed in the PDF-positive LN_v_s [28]. The *cry-Gal4–19* insertion was generated by jumping out the P element of the original *cry-Gal4* insertion [10]. It has a more restricted expression pattern than the previously described *cry-Gal4–39* insertion [11]. The expression patterns of *cry-Gal4–19* and *Mai179-Gal4* (see also [7]) are described in Figure S1. We used the *pdf-Gal80* line 96A, which contains two insertions and completely abolishes *pdf-Gal4*-driven expression in the PDF-positive LN_v_s [8].

### Behavioral analysis.

Experiments were carried out with 1–7-d-old flies at 20 °C in *Drosophila* activity monitors (TriKinetics) as previously described [38]. Light was provided by standard, white-fluorescent low-energy bulbs. Light intensity at fly level was in the range of 300–1,000 μW/cm^2^, depending on the position of the monitor in the incubator. For LL and DD analysis, flies were first entrained in 12 h:12 h LD cycles during at least 4 d, and activity data were analyzed for 6 d, starting from the second day in DD or in LL. Under these LL conditions, *cry^b^* mutants displayed robust activity rhythms, and no split rhythms could be observed. Data analysis was done with the FaasX 0.9.8 software, which is derived from the Brandeis Rhythm Package. FaasX runs on Apple Macintosh OSX and is freely available upon request. Rhythmic flies were defined by χ^2^ periodogram analysis with the following criteria (filter ON): power ≥ 20, width ≥ 2 h, with selection of 24 h ± 6 h upon period value. Power and width are the height and width of the periodogram peak, respectively, and give the significance of the calculated period. Actograms represent absolute activity levels for each 0.5-h interval, averaged over groups of flies of a given genotype. The hash density of the actogram (number of activity events per hash) varies from 15 to 35, according to the activity level of the genotype. This allows the comparison of activity profiles between genotypes that display very different activity levels. Mean daily activity (number of events per 0.5 h ± standard error of the mean [s.e.m.]) is calculated over the whole period of DD or LL, and is reported in Tables 1, 2, and S1 for all genotypes. All behavioral experiments were reproduced two or three times with very similar results.

### Immunolabelings.

All experiments were done on whole-mounted adult brains. GFP reporter expression, anti-PER, anti-CRY, and anti-PDF labeling was done as previously described [11,20]. Fluorescence signals were analyzed with a Zeiss Axioplan2 epifluorescence microscope equipped with a SPOT2 (Diagnostic Instruments) digital camera. Fluorescence intensity was quantified from digital images with the ImageJ software. We applied the formula: *I* = 100 × (*S* − *B*)/*B*, that gives the fluorescence percentage above background (where *S* is the fluorescence intensity, and *B* is the average intensity of the region adjacent to the positive cell). Confocal imaging was performed on a Leica SP2 microscope. Stacks of approximately 20 images were obtained, which spanned the breadth of the brain between the LN_v_s (posterior) and the DN1s (anterior). Maximum intensity projections were generated from such stacks.

## Supporting Information

Figure S1 Characterization of the *Mai179-Gal4* and *cry-Gal19* Expression Patterns Brains were dissected in LD conditions at ZT0. (A–D) *Mai179-Gal4* driven GFP expression is detected in the four PDF-positive s-LN_v_s, the fifth s-LN_v_, a small number of l-LN_v_s (weak), three LN_d_s, and two DN1s (weak) plus other non-clock neuronal groups [2].
(A) Epifluorescence images. GFP and anti-PDF staining identify *Mai179-Gal4*–expressing PDF-positive and PDF-negative LN_v_s.
(B–D) Confocal projections. *Mai179-Gal4*–driven GFP and PER expression in *per^0^* flies. PER is strongly expressed in the five s-LN_v_s and three LN_d_s. Highly variable PER expression could be detected in a pair of DN1s ( [C and D], 0.6–0.7 labeled DNs per hemisphere on average). An even weaker PER expression was observed in the DN1 neurons in LL (0.1–0.2 labeled cells per hemisphere; unpublished data).
(E) Confocal projections. *cry-Gal4–19*–driven GFP and PER expression in *per^0^* flies. GFP is detected in the five s-LN_v_s, three to six LN_d_s, and two DN1s. PER is expressed in the five s-LN_v_s, some l-LN_v_s (weak), three LN_d_s *(Mai179-Gal4–*positive; unpublished data), and two DN1s. A noncycling expression was observed in the DN1 neurons in LL (unpublished data). From their anterior and very dorsal position, the two DN1s seen with both drivers correspond well with the DN1a described in [5]. Stars indicate nonspecific labeling.
Scale bars indicate 40 μm in (B and E), and 20 μm in (C and D).
(2.0 MB PDF)

Figure S2 Oscillator-Autonomous Inhibition of PER Accumulation by CRY in LL PER and PDF immunoreactivity is shown after 48 h in LL. Representative half-brains of flies with CRY in all clock neuron groups (wild-type control [A]), in none (*cry^b^* control [B]), in the LN-MO (PDF-positive LN_v_s only [C]), in all PDF-negative groups (D and E), or in the LN-EO only (F) are shown. The genotypes in (C–F) correspond to those of Figure 1B–1E. The largest LN_d_ in (B and C) appears to correspond to the LN_d_^M*^ characterized with the help of the *Mai179-Gal4* driver, whereas the other three are likely to be the LN_d_^O^s, which are labeled at all time points (see Figure 1). DNs can be seen in the three genotypes in which these cells are devoid of CRY (B, C, and F). However, in line with previous results [27], we observed no PER cycling in these cells (unpublished data). Scale bar indicates 40 μm.
(1.2 MB PDF)

Figure S3 PER and PDF Immunoreactivity in the LNs of PER Rescued Flies in LL or DD Brains were dissected after 52 to 56 h of LL (A) or DD (B–D). The genotypes in (A–D) correspond to those of Figure 4A–4D.
(A) *Mai179-Gal4/pdf-Gal80* drives PER expression in three LN_d_s (one LN_d_^M*^ + two LN_d_^M^s; see Figure S1) and in the fifth PDF-negative s-LN_v_ in LL.
(B) In DD, *Mai179-Gal4/pdf-Gal80* drives PER expression in three LN_d_s (one LN_d_^M*^ + two LN_d_^M^s), but not in the fifth PDF-negative s-LN_v_. In DD, *Mai179-Gal4* expression is in fact undetectable in all five s-LN_v_s (P. Cusumano and F. Rouyer, unpublished data; see [7]).
(C) *cry-Gal4–19/pdf-Gal80* drives PER expression in three LN_d_s (one LN_d_^M*^ + two LN_d_^M^s), in the fifth PDF-negative s-LN_v_ and two DN1s (unpublished data) in DD.
(D) *pdf-Gal4* drives PER expression in the four PDF-positive s-LN_v_s and the l-LN_v_s (out of focus in the picture) in DD (see [7]).. Black boxes separate regions taken from different focal planes. Scale bar : 20 μm.
AR, arrhythmic flies; R, rhythmic flies (see Table 1).
(1.0 MB PDF)

Figure S4 PER Oscillations in *cry^b^ pdf^0^* Flies in LL Fly entrainment and PER quantification were performed as described in Figure 1. Brains were dissected during the third day in LL. PER cycling in the PDF-negative fifth s-LN_v_ was out of phase with PER cycling in the PDF-expressing s-LN_v_s, in agreement with a peak around Zeitgeber time (ZT)12 in LD conditions (P. Cusumano and F. Rouyer, unpublished data). This fits with the phase-shifted activity bout of the *cry^b^ pdf^0^* flies in LL, compared to *cry^b^* flies (see Figures 3 and 5). PER oscillations in the fifth s-LN_v_ were therefore expected to peak around circadian time (CT)57–58 in the third day of LL if the EO oscillator runs with a 22.8-h period (see Table 2). The observed peak of PER was indeed at CT58, whereas the PDF-expressing LN_v_s showed robust 24-h PER oscillations. In agreement with the *cry^b^* data (see Figure 2A), PER oscillations were similarly phased in the fifth s-LN_v_ and the LN_d_^M^s + LN_d_^M*^, although oscillations were broader and of lower amplitude in the LN_d_s so that it was difficult to distinguish between the LN_d_^M^s and LN_d_^M*^. As in *cry^b^* flies, no oscillations were observed in the LN_d_^O^s.
(121 KB PDF)

Table S1 Locomotor Activity Rhythms of Control Flies The mean values of circadian period (h), associated powers (see Materials and Methods), and activities (number of events per 0.5 h) are given ± s.e.m.
(62 KB PDF)

## Abbreviations

CRY: cryptochrome
DD: dark–dark (constant darkness)
DN: dorsal neuron
EO: evening oscillator
GFP: green fluorescent protein
LD: light–dark
LL: light–light (constant light)
l-LN_v_: large ventral lateral neuron
LN: lateral neuron
LN_d_: dorsal lateral neuron
LN-EO: the evening oscillator residing in pigment-dispersing factor–negative lateral neurons
LN-MO: the morning oscillator residing in pigment-dispersing factor–positive lateral neurons
MO: morning oscillator
PDF: pigment-dispersing factor
PER: PERIOD
RNAi: RNA interference
s.e.m.: standard error of the mean
s-LN_v_: small ventral lateral neuron

## References

[pbio-0050315-b001] Saunders DS, Steel CGH, Vafopoulou X, Lewis RD (2002). Insect clocks. 3rd Edition.

[pbio-0050315-b002] Dunlap JC, Loros JJ, DeCoursey PJ (2004). Chronobiology. Biological timekeeping.

[pbio-0050315-b003] Pittendrigh C, Daan S (1976). A functional analysis of circadian pacemakers in nocturnal rodents. V. Pacemaker structure: a clock for all seasons. J Comp Physiol A.

[pbio-0050315-b004] Hall JC (2003). Genetics and molecular biology of rhythms in Drosophila and other insects. Adv Genet.

[pbio-0050315-b005] Shafer OT, Helfrich-Forster C, Renn SC, Taghert PH (2006). Reevaluation of Drosophila melanogaster's neuronal circadian pacemakers reveals new neuronal classes. J Comp Neurol.

[pbio-0050315-b006] Helfrich-Forster C, Shafer OT, Wulbeck C, Grieshaber E, Rieger D (2007). Development and morphology of the clock-gene-expressing lateral neurons of Drosophila melanogaster. J Comp Neurol.

[pbio-0050315-b007] Grima B, Chélot E, Xia R, Rouyer F (2004). Morning and evening peaks of activity rely on different clock neurons of the Drosophila brain. Nature.

[pbio-0050315-b008] Stoleru D, Peng P, Agosto J, Rosbash M (2004). Coupled oscillators control morning and evening locomotor behavior of Drosophila. Nature.

[pbio-0050315-b009] Stoleru D, Peng Y, Nawathean P, Rosbash M (2005). A resetting signal between Drosophila pacemakers synchronizes morning and evening activity. Nature.

[pbio-0050315-b010] Emery P, Stanewsky R, Helfrich-Förster C, Emery-Le M, Hall JC (2000). Drosophila CRY is a deep brain circadian photoreceptor. Neuron.

[pbio-0050315-b011] Klarsfeld A, Malpel S, Michard-Vanhée C, Picot M, Chélot E (2004). Novel features of cryptochrome-mediated photoreception in the brain circadian clock of Drosophila. J Neurosci.

[pbio-0050315-b012] Ceriani MF, Darlington TK, Staknis D, Mas P, Petti AA (1999). Light-dependent sequestration of TIMELESS by CRYPTOCHROME. Science.

[pbio-0050315-b013] Naidoo N, Song W, Hunter-Ensor M, Sehgal A (1999). A role for the proteasome in the light response of the timeless clock protein. Science.

[pbio-0050315-b014] Busza A, Emery-Le M, Rosbash M, Emery P (2004). Roles of the two Drosophila CRYPTOCHROME structural domains in circadian photoreception. Science.

[pbio-0050315-b015] Dissel S, Codd V, Fedic R, Garner KJ, Costa R (2004). A constitutively active cryptochrome in Drosophila melanogaster. Nat Neurosci.

[pbio-0050315-b016] Emery P, So WV, Kaneko M, Hall JC, Rosbash M (1998). CRY, a Drosophila clock and light-regulated cryptochrome, is a major contributor to circadian rhythm resetting and photosensitivity. Cell.

[pbio-0050315-b017] Stanewsky R, Kaneko M, Emery P, Beretta B, Wager-Smith K (1998). The cryb mutation identifies cryptochrome as a circadian photoreceptor in Drosophila. Cell.

[pbio-0050315-b018] Helfrich-Förster C, Winter C, Hofbauer A, Hall JC, Stanewsky R (2001). The circadian clock of fruit flies is blind after elimination of all known photoreceptors. Neuron.

[pbio-0050315-b019] Helfrich-Förster C, Edwards T, Yasuyama K, Wisotzky B, Schneuwly S (2002). The extraretinal eyelet of Drosophila: development, ultrastructure and putative circadian function. J Neurosci.

[pbio-0050315-b020] Malpel S, Klarsfeld A, Rouyer F (2002). Larval optic nerve and adult extra-retinal photoreceptors sequentially associate with the clock neurons during Drosophila brain development. Development.

[pbio-0050315-b021] Rieger D, Stanewsky R, Helfrich-Förster C (2003). Cryptochrome, compound eyes, H-B eyelets and ocelli play different roles in the entrainment and masking pathway of the locomotor activity rhythm in the fruit fly Drosophila melanogaster. J Biol Rhythms.

[pbio-0050315-b022] Aschoff J, Aschoff J (1981). Free running and entrained circadian rhythms. Biological rhythms. Volume 4, Handbook of behavioral neurobiology.

[pbio-0050315-b023] Emery P, Stanewsky R, Hall JC, Rosbash M (2000). A unique circadian-rhythm photoreceptor. Nature.

[pbio-0050315-b024] Peschel N, Veleri S, Stanewsky R (2006). Veela defines a molecular link between Cryptochrome and Timeless in the light-input pathway to Drosophila's circadian clock. Proc Natl Acad Sci U S A.

[pbio-0050315-b025] Koh K, Zheng X, Sehgal A (2006). JETLAG resets the Drosophila circadian clock by promoting light-induced degradation of TIMELESS. Science.

[pbio-0050315-b026] Yoshii T, Funada Y, Ibuki-Ishibashi T, Matsumoto A, Tanimura T (2004). Drosophila cry(b) mutation reveals two circadian clocks that drive locomotor rhythm and have different responsiveness to light. J Insect Physiol.

[pbio-0050315-b027] Rieger D, Shafer OT, Tomioka K, Helfrich-Forster C (2006). Functional analysis of circadian pacemaker neurons in Drosophila melanogaster. J Neurosci.

[pbio-0050315-b028] Renn SC, Park JH, Rosbash M, Hall JC, Taghert PH (1999). A pdf neuropeptide gene mutation and ablation of PDF neurons each cause severe abnormalities of behavioral circadian rhythms in Drosophila. Cell.

[pbio-0050315-b029] Kaneko M (1998). Neural substrates of Drosophila rhythms revealed by mutants and molecular manipulations. Curr Opin Neurobiol.

[pbio-0050315-b030] Bergmann A, Agapite J, McCall K, Steller H (1998). The Drosophila gene hid is a direct molecular target of Ras-dependent survival signaling. Cell.

[pbio-0050315-b031] Lin Y, Stormo GD, Taghert PH (2004). The neuropeptide PDF coordinates pacemaker interactions in the Drosophila circadian system. J Neurosci.

[pbio-0050315-b032] Murad A, Emery-Le M, Emery P (2007). A subset of dorsal neurons modulates circadian behavior and light responses in Drosophila. Neuron.

[pbio-0050315-b033] Stoleru D, Nawathean P, Fernandez Mde L, Menet JS, Ceriani MF (2007). The Drosophila circadian network is a seasonal timer. Cell.

[pbio-0050315-b034] Peng P, Stoleru D, Levine JD, Hall JC, Rosbash M (2003). Drosophila free-running rhythms require intercellular communication. PLoS Biol.

[pbio-0050315-b035] Blanchardon E, Grima B, Klarsfeld A, Chélot E, Hardin PE (2001). Defining the role of Drosophila lateral neurons in the control of circadian activity and eclosion rhythms by targeted genetic ablation and PERIOD protein overexpression. Eur J Neurosci.

[pbio-0050315-b036] Rubin EB, Shemesh Y, Cohen M, Elgavish S, Robertson HM (2006). Molecular and phylogenetic analyses reveal mammalian-like clockwork in the honey bee (Apis mellifera) and shed new light on the molecular evolution of the circadian clock. Genome Res.

[pbio-0050315-b037] Yuan Q, Metterville D, Briscoe AD, Reppert SM (2007). Insect cryptochromes: gene duplication and loss define diverse ways to construct insect circadian clocks. Mol Biol Evol.

[pbio-0050315-b038] Klarsfeld A, Leloup JC, Rouyer F (2003). Circadian rhythms of locomotor activity in Drosophila. Behav Processes.

