# Supplementary figures and images for: Light Activates Output from Evening Neurons and Inhibits Output from Morning Neurons in the Drosophila Circadian Clock
Source: PLoS Biol. 2007 Nov 27;5(11):e315. doi: 10.1371/journal.pbio.0050315 (PMC2229858; doi:10.1371/journal.pbio.0050315)

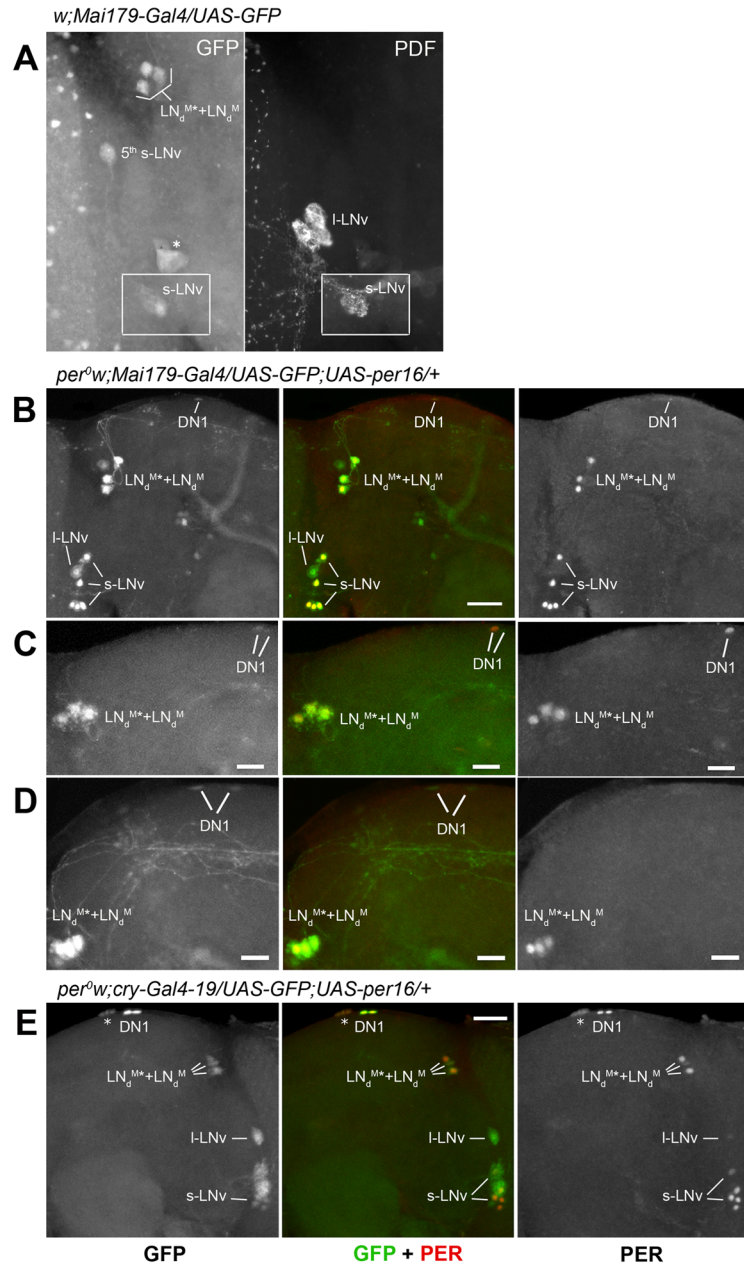

Supplementary Fig. 1 : Characterization of the Mai179-Gal4 and cry-Gal4-19 expression patterns

Supplement: Figure S1 — Brains were dissected in LD conditions at ZT0. (A–D) Mai179-Gal4 driven GFP expression is detected in the four PDF-positive s-LNvs, the fifth s-LNv, a small number of l-LNvs (weak), three LNds, and two DN1s (weak) plus other non-clock neuronal groups [2]. (A) Epifluorescence images. GFP and anti-PDF staining identify Mai179-Gal4–expressing PDF-positive and PDF-negative LNvs. (B–D) Confocal projections. Mai179-Gal4–driven GFP and PER expression in per0 flies. PER is strongly expressed in the five s-LNvs and three LNds. Highly variable PER expression could be detected in a pair of DN1s ( [C and D], 0.6–0.7 labeled DNs per hemisphere on average). An even weaker PER expression was observed in the DN1 neurons in LL (0.1–0.2 labeled cells per hemisphere; unpublished data). (E) Confocal projections. cry-Gal4–19–driven GFP and PER expression in per0 flies. GFP is detected in the five s-LNvs, three to six LNds, and two DN1s. PER is expressed in the five s-LNvs, some l-LNvs (weak), three LNds (Mai179-Gal4–positive; unpublished data), and two DN1s. A noncycling expression was observed in the DN1 neurons in LL (unpublished data). From their anterior and very dorsal position, the two DN1s seen with both drivers correspond well with the DN1a described in [5]. Stars indicate nonspecific labeling. Scale bars indicate 40 μm in (B and E), and 20 μm in (C and D). (2.0 MB PDF) [file pbio.0050315.sg001.pdf]

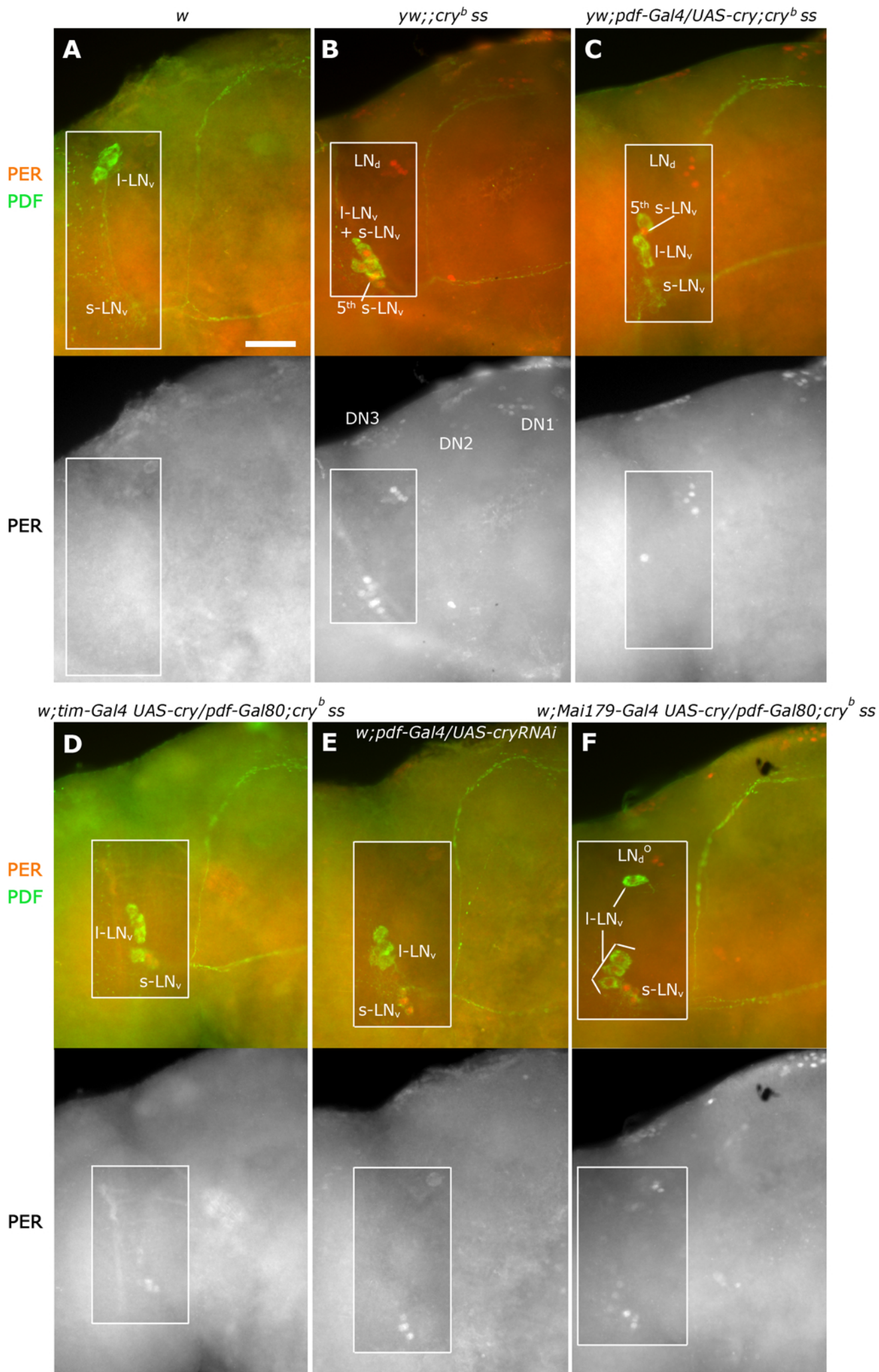

Supplementary Fig. 2 : Oscillator-autonomous inhibition of PER accumulation by CRY in LL

Supplement: Figure S2 — PER and PDF immunoreactivity is shown after 48 h in LL. Representative half-brains of flies with CRY in all clock neuron groups (wild-type control [A]), in none (cryb control [B]), in the LN-MO (PDF-positive LNvs only [C]), in all PDF-negative groups (D and E), or in the LN-EO only (F) are shown. The genotypes in (C–F) correspond to those of Figure 1B–1E. The largest LNd in (B and C) appears to correspond to the LNd M* characterized with the help of the Mai179-Gal4 driver, whereas the other three are likely to be the LNd Os, which are labeled at all time points (see Figure 1). DNs can be seen in the three genotypes in which these cells are devoid of CRY (B, C, and F). However, in line with previous results [27], we observed no PER cycling in these cells (unpublished data). Scale bar indicates 40 μm. (1.2 MB PDF) [file pbio.0050315.sg002.pdf]

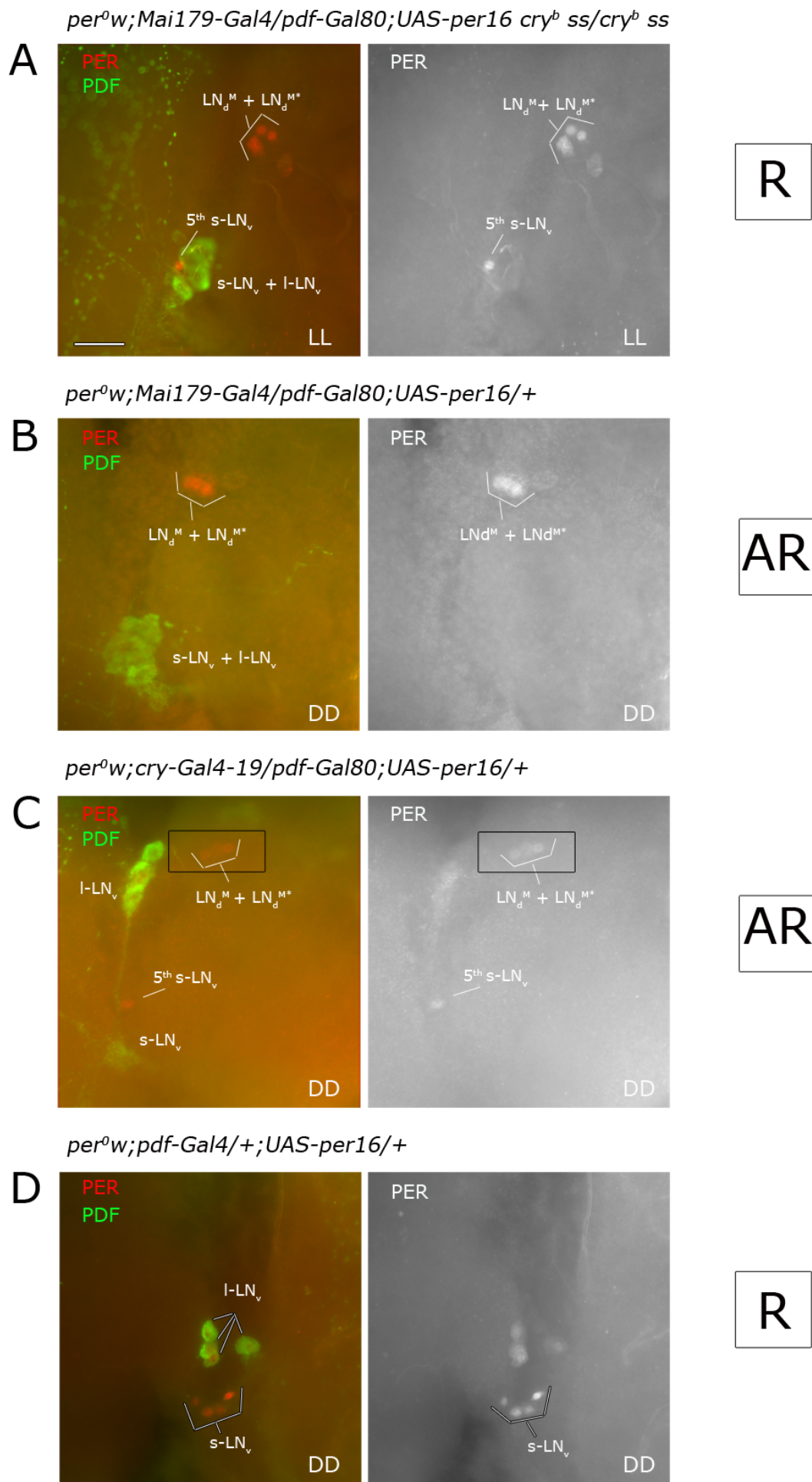

Supplementary Fig. 3: PER immunoreactivity in the LNs of PER-rescued flies in LL or DD

Supplement: Figure S3 — Brains were dissected after 52 to 56 h of LL (A) or DD (B–D). The genotypes in (A–D) correspond to those of Figure 4A–4D. (A) Mai179-Gal4/pdf-Gal80 drives PER expression in three LNds (one LNd M* + two LNd Ms; see Figure S1) and in the fifth PDF-negative s-LNv in LL. (B) In DD, Mai179-Gal4/pdf-Gal80 drives PER expression in three LNds (one LNd M* + two LNd Ms), but not in the fifth PDF-negative s-LNv. In DD, Mai179-Gal4 expression is in fact undetectable in all five s-LNvs (P. Cusumano and F. Rouyer, unpublished data; see [7]). (C) cry-Gal4–19/pdf-Gal80 drives PER expression in three LNds (one LNd M* + two LNd Ms), in the fifth PDF-negative s-LNv and two DN1s (unpublished data) in DD. (D) pdf-Gal4 drives PER expression in the four PDF-positive s-LNvs and the l-LNvs (out of focus in the picture) in DD (see [7]).. Black boxes separate regions taken from different focal planes. Scale bar : 20 μm. AR, arrhythmic flies; R, rhythmic flies (see Table 1). (1.0 MB PDF) [file pbio.0050315.sg003.pdf]

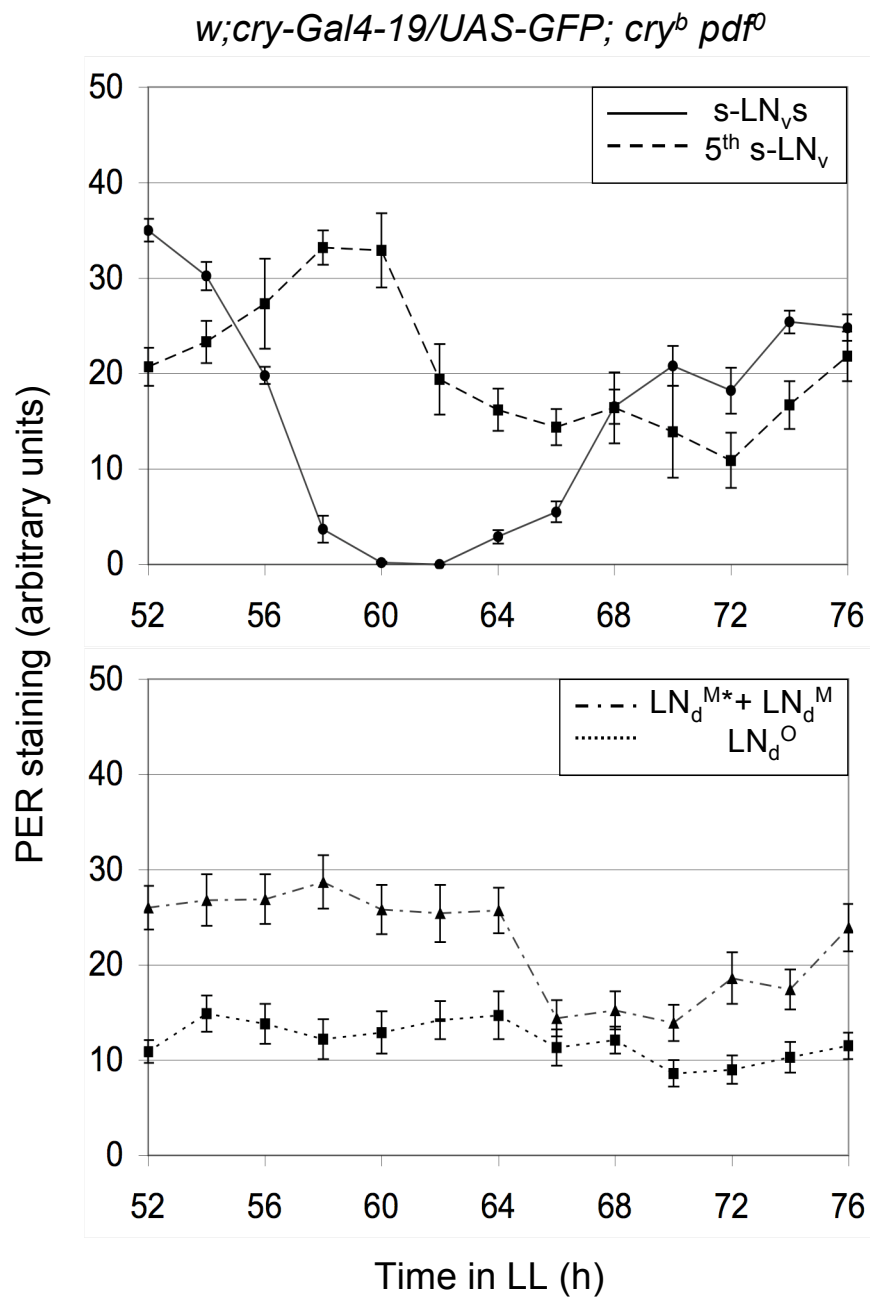

Suppl. Figure 4 : PER oscillations in *cry<sup>b</sup> pdf<sup>0</sup>* flies in constant light

Supplement: Figure S4 — Fly entrainment and PER quantification were performed as described in Figure 1. Brains were dissected during the third day in LL. PER cycling in the PDF-negative fifth s-LNv was out of phase with PER cycling in the PDF-expressing s-LNvs, in agreement with a peak around Zeitgeber time (ZT)12 in LD conditions (P. Cusumano and F. Rouyer, unpublished data). This fits with the phase-shifted activity bout of the cryb pdf0 flies in LL, compared to cryb flies (see Figures 3 and 5). PER oscillations in the fifth s-LNv were therefore expected to peak around circadian time (CT)57–58 in the third day of LL if the EO oscillator runs with a 22.8-h period (see Table 2). The observed peak of PER was indeed at CT58, whereas the PDF-expressing LNvs showed robust 24-h PER oscillations. In agreement with the cryb data (see Figure 2A), PER oscillations were similarly phased in the fifth s-LNv and the LNd Ms + LNd M*, although oscillations were broader and of lower amplitude in the LNds so that it was difficult to distinguish between the LNd Ms and LNd M*. As in cryb flies, no oscillations were observed in the LNd Os. (121 KB PDF) [file pbio.0050315.sg004.pdf]
