# Supplementary material for: Light Activates Output from Evening Neurons and Inhibits Output from Morning Neurons in the Drosophila Circadian Clock
Source: PLoS Biol. 2007 Nov 27;5(11):e315. doi: 10.1371/journal.pbio.0050315 (PMC2229858; doi:10.1371/journal.pbio.0050315)
Supplement: Table S1 — The mean values of circadian period (h), associated powers (see Materials and Methods), and activities (number of events per 0.5 h) are given ± s.e.m. (62 KB PDF) [file pbio.0050315.st001.pdf]

Genotype	Total flies (n)	Rhythmic flies (%)	Period (h)			Power			Activity		
yw;pdf-Gal4/+;cry^b ss	31	84%	25.2	±	0.1	91	±	7	13	±	1
w:cry-Gal4-19/+:cry^b ss	16	69%	25.4	±	0.3	59	±	8	23	±	3
yw;pdf-Gal80/CyO;cry^b ss	16	100%	24.9	±	0.1	149	±	8	10	±	1
yw:UAS-cry/+:cry^b ss	16	100%	25.5	±	0.1	111	±	9	17	±	1
yw;;UAS-per16cryb/cry^{b}ss	62	98%	24.8	±	0.1	100	±	4	12	±	1
yw:tim-Gal4/UAS-GFP:cry^b ss	47	91%	24.9	±	0.1	83	±	5	14	±	2
yw:pdf-Gal4/UAS-GFP:cry^b ss	31	90%	25.0	±	0.1	100	±	8	13	±	1
w:Mai179-Gal4/UAS-GFP:cry^b ss	70	93%	25.0	±	0.1	86	±	4	15	±	1
w:cry-Gal4-19/UAS-GFP:cry^b ss	44	89%	25.1	±	0.2	99	±	7	16	±	1

Supplementary Table 1 :

Locomotor activity rhythms of control flies. The mean values of circadian period (h), associated powers (see Methods) and activities (number of events per 0,5h) are given ± s.e.m.
